# Association of tissue oxygen saturation levels with skeletal muscle injury in the critically ill

**DOI:** 10.1038/s41598-024-55118-1

**Published:** 2024-02-27

**Authors:** Ryuji Sugiya, Shinichi Arizono, Yuji Higashimoto, Yuta Kimoto, Masashi Shiraishi, Hiroki Mizusawa, Yuichi Tawara, Hironori Shigeoka, Jan Bakker, Koichiro Shinozaki

**Affiliations:** 1 Department of Rehabilitation Medicine, Faculty of Medicine, Kindai University, Osaka, Japan; 2 School of Rehabilitation Science, Seirei Christopher University, Shizuoka, Japan; 3 Department of Emergency Medicine, Faculty of Medicine, Kindai University, 377-2 Onohigashi, Osakasayama, Osaka 589-8511 Japan; 4 Department of Intensive Care, Erasmus MC University Medical Center, Rotterdam, The Netherlands; 5 Department of Pulmonology and Critical Care, Columbia University Medical Center, New York, USA; 6 NYU School of Medicine Langone, New York, USA; 7 Departamento de Medicina Intensiva, Facultad de Medicina, Pontificia Universidad Católica de Chile, Santiago, Chile; 8 Feinstein Institutes for Medical Research, Manhasset, NY USA

**Keywords:** Tissue oxygen saturation, Ultrasonography, Medical Research Council (MRC) scale, Near-infrared spectroscopy, Rectus femoris, Cross-sectional area, Medical research, Risk factors

## Abstract

This study aimed to investigate the association between the level of tissue oxygen saturation (StO_2_) and quadriceps/skeletal muscle dysfunction, measured using the Medical Research Council (MRC) scale and ultrasonography, in critically ill patients. Thirty-four patients hospitalized at the Critical Care Medicine Center of Kindai University Hospital, between January 2022 and March 2023, were enrolled in this study. The StO_2_ of the quadriceps muscle was measured via near-infrared spectroscopy. Muscle atrophy was measured by the thickness, cross-sectional area (CSA), and echo intensity of the rectus femoris (RF). These values were evaluated every alternate day until 13 days after admission or until discharge, whichever occurred first. Muscle weakness was assessed using the sum score of the MRC scale (MRC-SS), with the patient sitting at bedside. The mean age of the patients was 67.3 ± 15.3 years, and 20 (59%) were men. Seven patients (21%) were admitted for trauma, and 27 (79%) were admitted for medical emergencies or others. The mean score for the MRC-SS was 51.0 ± 7.9 points. RF thickness and CSA significantly decreased after day 7 (*p* < 0.05). There were no significant changes in StO_2_ levels during hospitalization. However, there were positive correlations between the nadir StO_2_ during hospitalization and MRC-SS, and changes in RF thickness and CSA at discharge (r = 0.41, *p* = 0.03; r = 0.37, *p* = 0.03; and r = 0.35, *p* = 0.05, respectively). StO_2_ in the quadriceps muscle may be useful for predicting muscle atrophy and dysfunction in patients with critical illnesses.

In recent years, intensive care unit-acquired muscle weakness (ICU-AW) has been recognized as a disorder frequent complication of critical illness^1^. ICU-AW is found in approximately 50% of adult ICU patients^2^ associated with acute disability and worsened long-term prognosis^3,4^.

There are numbers of risk factors associated with the development of ICU-AW, e.g., sepsis, multiple organ failure, mechanical ventilation, exposure to glucocorticoids or neuromuscular blocking agents, and poor glycemic control^2^. Systemic inflammation is a key driver for muscle protein loss^5^. Pro-inflammatory cytokine levels, such as of interleukin-6 (IL-6), have been associated with volume loss of skeletal muscle volume due to their catabolic effects^6^. However, cytokine measurements are not convenient and frequent measurements are not feasible in many ICUs.

Near-infrared spectroscopy (NIRS) is a noninvasive technique to measure the amount of light absorbed by hemoglobin that varies with the level of oxygenation. NIRS uses the absorption of infrared light at three specific wavelengths (680, 810, and 830 nm) to assess oxyhemoglobin (oxy-Hb) and deoxyhemoglobin (deoxy-Hb) in order to estimate the tissue oxygen saturation (StO_2_). Several studies reported the effectiveness of StO_2_ measurements in patients with sepsis, where it has been suggested that muscle StO_2_ can be a strong predictor of mortality^7–9^.

Therefore, we hypothesized that systematic inflammation would increase catabolism, due to increased levels of pro-inflammatory cytokines and thus may change StO_2_. As a result, muscle strength and mass could decrease in patients showing low StO_2_. However, relationship between StO_2_ and muscle atrophy has barely elucidated. We hypothesized that muscle atrophy in ICU-AW would be correlated with increased muscle oxygen consumption and/or impaired perfusion. The purpose of this study was to demonstrate that StO_2_ variables as a marker of muscle atrophy and ICU-AW.

## Methods

### Study design and participants

This was a single-center prospective cohort study of critically ill patients admitted to the critical care medical center at the tertiary care teaching hospital (Kindai University Hospital) between January 2022 and March 2023.

The demographic and clinical data of all the patients, including medications, sedative agents, and ventilator settings were recorded. A severity of disease was evaluated by using the Acute Physiology and Chronic Health Evaluation (APACHE) II scoring and the Sequential Organ Failure Assessment scoring system^10,11^.

This study was conducted at the tertiary care teaching hospital and we enrolled any critically ill patients admitted to the critical care medical center, in which our patients included: acute abdomen, sepsis, trauma, poison, acute lung disease, soft tissue infection, burn, cardiac arrest, and metabolic crisis. The study inclusion criteria were aged 18 years or older and able to provide written informed consent from the patient or next of kin. We used a convenient sample of our patients due to the nature of the exploratory phase of this study.

The exclusion criteria for this study were as follows: pregnancy, current imprisonment, cognitive impairment, a determination of instability by the clinical team, COVID-19, poor pre-hospital functional status (modified Rankin Scale ≥ 3^12^), stroke or neuromuscular disease, difficulty in measuring Medical Research Council (MRC) scale, finger and thigh anatomical anomalies or diseases that interfered with attaching a pulse oximetry sensor and NIRS probe. Patients who could not undergo MRC scale such as femur fracture were excluded. Patients with COVID-19 were excluded because we were not allowed to bring the instruments such as ultrasonography or NIRS into the patient`s room.

All methods were performed in accordance with relevant guidelines and regulations. The study protocol was approved by the IRB of the Kindai University Hospital. Written informed consent was obtained from the patients or their next kin. When the patients were admitted to the hospital, designated investigators received a written informed consent from the patient or his/her next kin. When we obtained a consent, we asked the patient or next of kin about which was the dominant hand. If the patients were alert and oriented, we explained them again before starting any measurements. Patients could be withdrawn from the study at any time.

### Primary outcome and secondly outcome

We investigated the relationship between low StO_2_ and MRC scale scores as the primary outcome. Additionally, relationship between low StO_2_ and muscle atrophy measured via ultrasonography as the secondary outcome.

### Assessment of NIRS measurements

**Figure 1:**
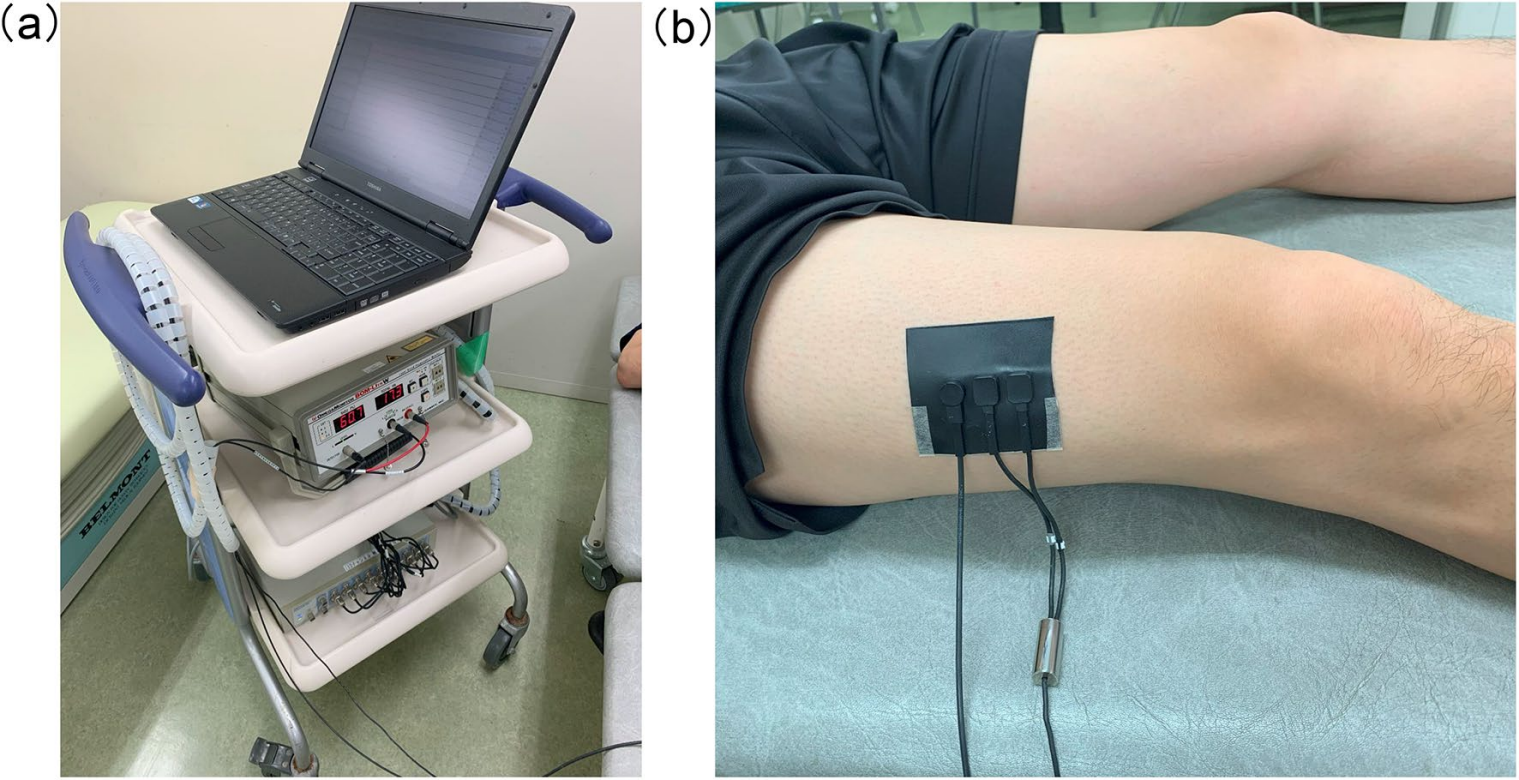
Measurement by the BOM-L1TRW laser tissue oximeter (OmegaWave, Inc., Tokyo, Japan). (**a**) A picture of the near-infrared spectroscopy (NIRS) system. (**b**) The NIRS probe was placed over the quadriceps muscle of the patient’s dominant side (1/3 of the distance from the greater trochanter to the middle border of the patella).

NIRS related variables were performed using a BOM-L1TRW laser tissue oximeter (OmegaWave, Inc., Tokyo, Japan) (Fig. 1). The NIRS probe was placed over the quadriceps muscle of the patient’s dominant side. This was identified by marking 1/3 of the distance from the greater trochanter to the middle border of the patella^13^. Measurements were performed while the patients were in a supine position, at rest. The values were recorded for at least one minute after stabilization, and the average value was calculated. We evaluated the patients’ oxy-Hb, deoxy-Hb, total hemoglobin, and StO_2_. All NIRS measurements were stored and analyzed using the LabChart 7.2 software (AD Instruments Ltd, Oxford, UK). Measurements were taken every alternate day after admission until day 13 or at discharge, whichever occurred first. For StO_2_, nadir, maximum, and average values during hospitalization were calculated for the analysis.

### Assessment of muscle atrophy and muscle weakness

Muscle atrophy of the rectus femoris (RF) was measured using the Xario 200 diagnostic ultrasound system (Canon Medical Systems Corporation, Tochigi, Japan) as reported by others^14^. The participants were assessed in the supine position with their knees in passive and neutral rotation. The probe was placed on the anterior part of the thigh, at 2/3 of an imaginary line connecting the anterior superior iliac spine and the midpoint of the proximal border of the patella^15^. Device settings (including gain and dynamic range) were kept constant between participants and across time points, with one experienced evaluator recording all the images. Gain and dynamic range were set at 70-dB and 80-dB, respectively. The depth of the image and focus might be different among patients. We adjusted the depth of the image so that the surface of the femur was seen as the landmark at 4–6 cm deep. The depth of the focus was also adjusted considering the difference of the subcutaneous fat thickness. Both settings were kept constant per patient across the study. A water-soluble transmission gel was applied to the ultrasound probe to allow acoustic contact without depressing the dermal surface. It could be difficult to obtain the entire cross-sectional area (CSA) in the ultrasound window especially for patients who had a good physique or using neuromuscular blocking agents. To solve this problem, we used “trapezoid scan” mode to expand the view of the linear transducer (Supplementary Fig. 1). By using this method, we were able to locate the whole CSA within the window and no patient was failed to the CSA measurement. The images were saved on an ultrasound hard drive and transferred to a computer using ImageJ software (National Institute of Health, Bethesda, MD, USA)^16^. We calculated the thickness, CSA, and echo intensity (EI) of the RF (Supplementary Fig. 1). The RF thickness was measured as the vertical distance between the superficial and deep fascia (cm). The CSA was marked along the hyperechoic fascia of the RF (cm^2^) using a freehand tracing tool. The EI was calculated via histogram analysis. Previous studies have reported that increased EI was associated with the infiltration of fat and fibrous tissues^17^. Measurements were performed every alternate day post-admission until day 13 and at discharge. Muscle atrophy measured by ultrasonography was evaluated and expressed as the percent change from day-1 measurement. Ultrasound images were taken three times per day and the values were averaged. In the field of ultrasonography measurements, it is seen as a problem that measurements accuracy would depend on the skill of observers. Therefore, in our study, all ultrasonography measurements were performed by the same/one investigator, who was blinded from the study results.

Muscle weakness was assessed using the sum of the MRC scale scores (MRC-SS)^18^. Using average score of the MRC scale is another way. However, because MRC-SS is more common, we selected MRC-SS. Therefore, we excluded patients, to whom we could not undergo MRC scale such as for patients with femur fracture. This was measured on the first day when the patients could sit in the edge-sitting position and follow five verbal commands^19^. We also assessed it at discharge. Patients with < 48 points were diagnosed with ICU-AWs.

### Assessment of the blood test measurement

The blood-test values were obtained from those at admission and at discharge. We extracted the data of: C-reactive protein (CRP); white blood cell (WBC); albumin (Alb); lactate dehydrogenase (LDH); and Lactate. IL-6 levels were measured only at admission by using an electrochemiluminescent immunoassay^20^, and logarithmic values were reported as a previous study^21^.

### Sample size

In line with a previous study^22^, we predicted that age, length of hospital stays, APACHE II score, and IL-6 level would be the candidate predictors. In addition, based on our hypothesis, we added StO_2_ as the potential predictor. Gender was also added to investigate a sex difference. Therefore, a total of 6 candidate predictors were chosen to be investigated by multivariate analysis. Five patients are required for each candidate predictor/factor and so at least 30 patients were needed. To account for possible dropout, 35 patients were recruited for this study.

### Statistical analysis

Values were presented as the mean ± standard deviation. Statistical significance was set at *p* < 0.05 for all analyses. Differences in blood test results between admission and discharge were compared using paired *t*-tests. Differences between each day (days 1, 3, 5, 7, 9, 11, 13, and at discharge) were analyzed by repeated measures analysis of variance (ANOVA) and Tukey analysis. Correlations between each factor, including NIRS and ultrasonography measurements, were assessed using Pearson’s correlation analysis. To elucidate the indicator(s) that might be associated with the MRC-SS, the stepwise procedure of a multiple regression analysis was performed by using a threshold of 0.05 for inclusion in and 0.10 for exclusion from the final model considering all possible combinations of main effects and interactions. We selected the predictor as below: age, length of hospital stay, APACHE II score, log IL-6. These factors have already been reported their relationship with muscle dysfunction^6,23,24^. We also included StO_2_ as the predictor based on our hypothesis. Moreover, gender difference was assessed. Before, we analyzed multiple regression analysis, we underwent the univariate analysis between muscle dysfunction and these related factors (supplemental data). We also compared the degree of association between IL-6 and StO_2_ by using the multiple regression analysis (supplemental data). As a sensitivity analysis, we performed multivariate analysis including sex as one of the confounders. Intra-observer reliability was assessed using intra-class correlation coefficients (ICC). ICC has been regarded within the framework of ANOVA and intra-observer reliability was calculated with one-way ANOVA. ICC is an index for the reliability of different measures. The ICC and their 95% confidence intervals were calculated based on mean rating (k = 3)^25^. All analyses were performed using SPSS 24.0 (IBM Corp., Armonk, NY, USA).

### Ethics approval

The study protocol was approved by the IRB of the Kindai University Faculty of Medicine (R4-001). Written informed consent was obtained from the patient or their next kin.

## Results

### Patient characteristics

**Figure 2:**
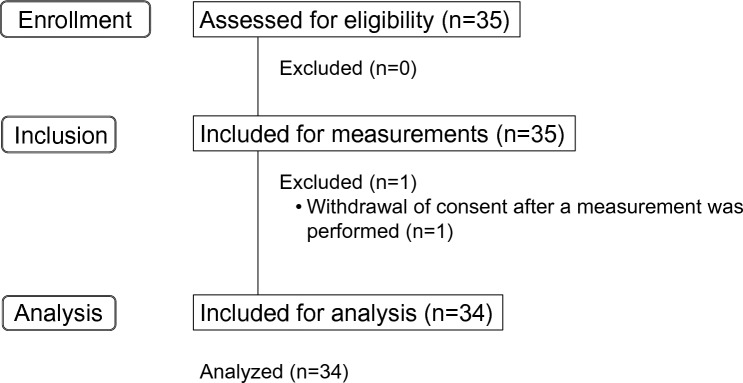
Flowchart of patient inclusion.

The participants’ characteristics are summarized in Table 1. A total of 34 patients were included in the analysis (Fig. 2). All the patients discharged or transferred to other hospital or home without moving to the general ward. The mean age of the patients was 67.3 ± 15.3 years, and 20 (59%) were male. Seven patients (21%) were admitted for trauma, and 27 (79%) were admitted for medical and other emergencies.

**Table 1:** Patient characteristics.

Variables	Critically ill patients (n = 34)
Age (years)	67.3 ± 15.3
Male, n (%)	20 (59)
Height (cm)	163.7 ± 6.9
Weight (kg)	63.1 ± 13.4
Body mass index (kg/m^2^)	23.5 ± 4.7
Length of hospital stay (days)	26.1 ± 21.0
APACHE II score (points)	17.5 ± 10.0
SOFA score (points)	5.0 ± 4.7
Charlson index (points)	1.3 ± 1.6
mRS (points)	0.6 ± 1.1
log IL-6 (pg/mL)	5.7 ± 2.7
Reasons for emergency transport	
Trauma, n (%)	7 (21)
Medical emergencies or others, n (%)	27 (79)
Diagnosis at admission	
Traumatic pneumothorax, n (%)	2 (6)
Head trauma, n (%)	5 (15)
Sepsis, n (%)	3 (9)
Acute pan-peritonitis, n (%)	3 (9)
Intestinal necrosis, n (%)	3 (9)
Mesenteric ischemia, n (%)	2 (6)
Gastrointestinal perforation, n (%)	5 (15)
Strangulation ileus, n (%)	2 (6)
Necrotizing fasciitis, n (%)	2 (6)
Acute renal failure, n (%)	1 (3)
Acute liver failure, n (%)	1 (3)
Drug overdose, n (%)	3 (9)
Burn, n (%)	1 (3)
Cardiac arrest, n (%)	1 (3)
Treatments	
Ventilator use, n (%)	15 (44)
Length of mechanical ventilation use (days)	12.4 ± 17.2
Length to start the mechanical ventilation (hours)	78.3 ± 101.5
Treatment with any glucocorticoids, n (%)	10 (29)
Treatment with any vasopressor, n (%)	14 (41)
Treatment with any neuromuscular blocker, n (%)	2 (6)

Values are shown as mean ± standard deviation, and number (%).

*APACHE* Acute Physiology and Chronic Health Evaluation, *SOFA* Sequential Organ Failure Assessment, *mRS* modified Rankin Scale, *IL-6* Interleukin-6.

The mean MRC-SS was 51.0 ± 7.9 points (Table 2). Six patients (18%) were diagnosed with ICU-AW. 27 patients underwent MRC scale assessment as 7 patients could not be assessed due to neurocognitive dysfunction. The mean MRC-SS has increased to 54.6 ± 5.4 points at discharge.

**Table 2:** Variables for MRC score and StO_2_ measurements.

Variables	Critically ill patients (n = 34)
Variables for MRC score	
MRC-SS (points)	51.0 ± 7.9
The number of patients underwent MRC score, n (%)	27 (79)
Diagnosed as ICU-AW, n (%)	6 (18)
Day of the first MRC assessment after admission (days)	7.9 ± 11.2
MRC-SS at discharge (points)	54.6 ± 5.4
Variables for StO_2_	
Nadir StO_2_ (%)	41.9 ± 9.4
Maximal StO_2_ (%)	52.4 ± 6.4
Average StO_2_ (%)	45.7 ± 6.3

Values are shown as mean ± standard deviation, and number (%).

*MRC-SS* Medical Research Council Sum Score, *ICU-AW* Intensive Care Unit-Acquired Weakness, *StO*_*2*_ tissue oxygen saturation.

### Changes in blood parameters

Changes in blood parameter values from admission to discharge are summarized in Table3. The patients’ CRP, WBC, albumin, LDH, and lactate levels significantly decreased at the time of discharge (*p* < 0.05).

**Table 3:** Changes in blood parameters at hospital admission and discharge.

Variables	At admission	At discharge	*p*-Value
CRP (mg/L)	8.8 ± 11.3	3.9 ± 4.3	0.02
WBC (× 10^3^/mm^3^)	11.9 ± 8.7	7.9 ± 4.7	0.01
Albumin (g/dL)	3.4 ± 1.1	2.6 ± 0.8	0.02
LDH (U/L)	330.5 ± 283.5	204.3 ± 88.9	< 0.01
Lactate (mg/dL)	25.0 ± 20.1	12.2 ± 6.2	< 0.01

Values are shown as the mean ± standard deviation.

*CRP* C-reactive protein, *WBC* white blood cell, *Alb* albumin, *LDH* lactate dehydrogenase.

### Change in variables related to NIRS and ultrasonography over time

**Figure 3:**
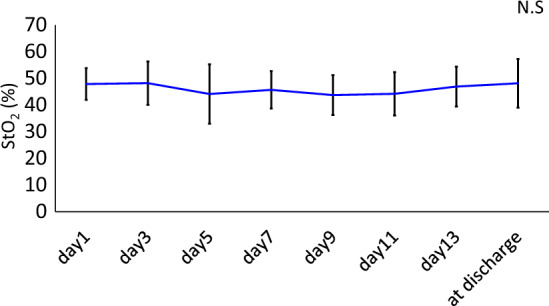
Change in StO_2_ over time during the hospitalization. StO_2_: tissue oxygen saturation.

The changes in the variables related to NIRS and ultrasonography are summarized in Table 4. The percentage changes in the RF thickness and CSA significantly decreased after hospital day 7 (*p* < 0.05). However, there was no significant change in EI. In addition, there were no significant changes in NIRS measurements and SpO_2_. The changes in StO_2_ over time are shown in Fig. 3. The StO_2_ values were calculated in detail. The nadir, maximum, and average StO_2_ were 41.9 ± 9.4, 52.4 ± 6.4, and 45.7 ± 6.3%, respectively (Table 2).

**Table 4:** Change over time in variables related to NIRS and US.

Variables	Day 1	Day 3	Day 5	Day 7	Day 9	Day 11	Day 13	At discharge
Percentage changes in values of US compared to admission								
Changes in RF thickness (%)	Ref	−5.3 ± 10.9	−8.7 ± 15.7	−16.0 ± 15.2 ^*a*^	−19.4 ± 21.3 ^*ab*^	−23.7 ± 18.8 ^*abc*^	−21.6 ± 18.7 ^*ab*^	−21.5 ± 15.6 ^*ab*^
Changes in RF CSA (%)	Ref	−4.1 ± 13.9	−6.6 ± 12.0	−17.2 ± 15.5 ^*a*^	−19.6 ± 15.8 ^*abc*^	−23.7 ± 16.1 ^*abc*^	−23.0 ± 14.2 ^*abc*^	−20.4 ± 17.1 ^*abc*^
Changes in RF EI (%)	Ref	−3.5 ± 16.3	−5.2 ± 14.2	−4.3 ± 12.9	−7.1 ± 13.9	−1.4 ± 16.8	−1.9 ± 14.2	−4.9 ± 13.8
Measurements of NIRS								
oxy-Hb (mM·mm)	13.1 ± 18.9	11.9 ± 11.9	10.9 ± 12.9	10.0 ± 12.3	1.4 ± 18.1	6.8 ± 14.5	14.1 ± 14.6	11.2 ± 15.1
deoxy-Hb (mM·mm)	14.0 ± 21.3	13.4 ± 12.4	12.0 ± 12.8	11.0 ± 14.4	1.8 ± 21.4	10.6 ± 9.0	14.9 ± 13.3	12.1 ± 14.9
total-Hb (mM·mm)	22.9 ± 24.2	22.4 ± 17.2	19.4 ± 16.9	18.2 ± 18.5	6.3 ± 28.3	17.4 ± 18.5	23.2 ± 14.2	18.8 ± 18.4
StO_2_ (%)	47.9 ± 6.0	48.2 ± 8.1	44.2 ± 11.1	45.7 ± 7.0	43.8 ± 7.5	44.3 ± 8.1	47.0 ± 7.5	48.2 ± 9.1
SpO_2_ (%)	97.9 ± 1.9	97.3 ± 2.1	97.4 ± 1.8	97.0 ± 1.7	96.7 ± 2.3	96.9 ± 1.6	96.8 ± 1.3	97.0 ± 1.5

*NIRS* Near-infrared spectroscopy*,* *US* ultrasonography*,* *CSA* cross-sectional area, *RF* rectus femoris, *EI* echo intensity, *NIRS* near-infrared spectroscopy, *oxy-Hb* oxyhemoglobin, *deoxy-Hb* deoxyhemoglobin, *total-Hb* total hemoglobin, *StO*_*2*_ tissue oxygen saturation, *SpO*_*2*_ oxygen saturation of the peripheral artery.

^*a*^*p* < 0.05 difference from day 1.

^*b*^*p* < 0.05 difference from day 3.

^*c*^*p* < 0.05 difference from day 5.

Relative changes of the RF thickness between days 1 and 7 were shown in Supplementary Fig. 2. A potential interaction of ultrasonography measurements by muscle edema has been discussed^26^.

### Variables related to MRC-SS and ultrasonography variables

**Figure 4:**
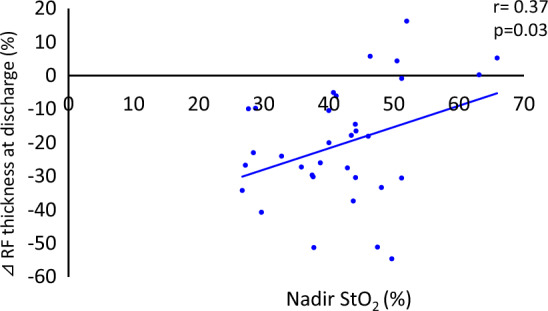
Correlation between nadir StO_2_ and percentage change in RF thickness at discharge (admission to ICU – > discharge from ICU). StO_2_: tissue oxygen saturation, RF: rectus femoris.

**Figure 5:**
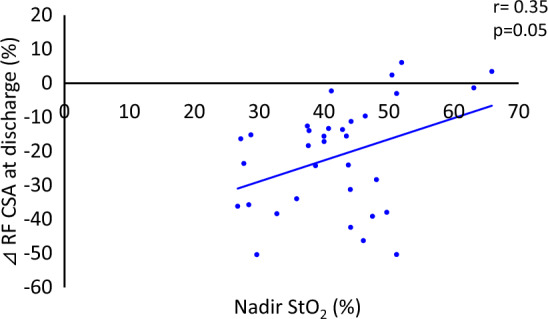
Correlation between nadir StO_2_ and percentage change in the rectus femoris CSA at discharge (admission to ICU – > discharge from ICU). StO_2_: tissue oxygen saturation, CSA: cross-sectional area.

Correlations between the variables are summarized in Table 5. The percentage change in RF thickness, CSA, and EI, from admission to discharge, were analyzed. The nadir StO_2_ was positively correlated with MRC-SS, changes in RF thickness, and CSA at discharge (r = 0.41, *p* = 0.03; r = 0.37, *p* = 0.03; and r = 0.35, *p* = 0.05, respectively), whereas the average StO_2_ and maximum StO_2_ had no significant correlation with any variables (Figs. 4 and 5). There was a negative correlation between log IL-6 and MRC-SS, and the percentage change in RF thickness and CSA (r = −0.53, *p* < 0.01; r = −0.57, *p* < 0.01; and r = −0.46, *p* = 0.01, respectively). The length of hospitalization was negatively correlated with the MRC-SS and the percentage change in RF CSA at discharge (r = −0.59, *p* < 0.01; r = −0.51, *p* < 0.01, respectively). Correlations between blood parameter values and other variables (MRC-SS, NIRS and ultrasonography measurements) are shown in Supplementary Table S1. There were no significant correlations among these values.

**Table 5:** Correlation between each StO_2_ measurement and other variables.

Variables	Coefficient correlation (r)				
	Nadir StO_2_	Average StO_2_	Maximum StO_2_	log IL-6	Length of hospital stay
MRC-SS (points)	0.41*	−0.28	−0.23	−0.53**	−0.59**
Changes in RF thickness (%)	0.37*	0.08	−0.05	−0.57**	−0.25
Changes in RF CSA (%)	0.35*	−0.11	−0.15	−0.46**	−0.51**
Changes in RF EI (%)	−0.10	−0.14	0.12	0.03	−0.12

*StO*_*2*_ tissue oxygen saturation, *MRC-SS* Medical Research Council sum score, *RF* rectus femoris, *CSA* cross-sectional area, *EI* echo intensity.

*p < 0.05, **p < 0.01.

### Factors associated with MRC-SS and ultrasonography variables

Multiple regression analysis was performed with the MRC-SS, change in RF thickness, and CSA (admission to ICU – > discharge from ICU) as dependent variables, and age, length of hospitalization, APACHE II score, log IL-6, and nadir StO_2_ as independent variables. Before the multiple regression analysis, we underwent the univariate analysis (Supplementary Table S2). As the result, all the independent factors above were significantly correlated with MRC-SS except age. There was no significant correlation between MRC-SS and ultrasonography measurements at admission (RF thickness and RF CSA). Therefore, we included the variables which were significant as the independent variables. Multiple regression analysis for MRC-SS was shown in Table 6. The results of multiple regression analysis showing the degree of associations compared between IL-6 and nadir StO_2_ were shown in Supplementary Table S3. Multiple regression analysis of changes in RF thickness and CSA were shown in Supplementary Tables S4 and S5. Predictive accuracy was lower when ultrasonography measurements were calculated as the dependent variable compared with MRC-SS.

**Table 6:** Multiple regression models for independent prediction of MRS-SS.

Variables	β	*p*-Value	Adjusted R^2^
Length of hospital stay (days)	−0.40	0.01	0.55
APACHE II score (points)	−0.45	< 0.01	
Nadir StO_2_ (%)	0.41	< 0.01	
Age (years)	†		
Gender	†		
log IL-6 (pg/mL)	†		

*MRC-SS* Medical Research Council Sum Score, *APACHE* Acute Physiology and Chronic Health Evaluation, *StO*_*2*_ tissue oxygen saturation.

^†^Variables not included in the model.

### The intra-rater reliability of the values measured by ultrasonography

The intra-rater reliability (intra-observer correlation) of the values measured by ultrasonography on admission was calculated as follows: RF thickness, ICC = 0.996 (0.992–0.998); RF CSA, ICC = 0.997 (0.994–0.997); and RF EI, ICC = 0.973 (0.953–0.986). These results indicate good reproducibility.

## Discussion

In this study, the nadir StO_2_ of the quadriceps muscle correlated with MRC-SS and the percentage change in RF thickness and CSA (admission to ICU – > discharge from ICU). To the best of our knowledge, this is the first study to demonstrate an association between muscle dysfunction and nadir StO_2_ levels in an ICU set-up. Similarly, log IL-6 also correlated with MRC-SS and the percentage change in RF thickness and CSA (admission to ICU – > discharge from ICU). However, when it was adjusted for APACHE II scores and length of hospitalization, StO_2_ was selected as the independent predictor, but IL-6 was not. These finding can help clinicians to predict the future impairments immediately after admission and take appropriate measures to prevent it.

Changes in StO_2_ indicate the alteration in muscle metabolism. Studies on exercise physiology demonstrated a decline in StO_2_ due to energy consumption during exercise^27^. StO_2_ is also measured in patients with sepsis, which is the most common inflammatory disease requiring ICU care^7–9^. Previous studies have reported that higher levels of inflammatory markers such as IL-6 are significantly associated with lower skeletal muscle strength and mass due to the catabolic effects^28^. We hypothesized that systemic inflammation increases metabolism in skeletal muscles, resulting in increased oxygen demand. As the result, there was a significant correlation between StO^2^ and MRC-SS which was the primary outcome of our study. The nadir StO_2_ was associated with MRC-SS and percentage changes in RF thickness and CSA (admission to ICU – > discharge from ICU), but not with average and maximum StO_2_. Furthermore, in the present study, length of hospitalization, APACHE II score, and nadir StO_2_ were significantly associated with MRC-SS. Several studies have reported that an occurrence of ICU-AW is linked with higher APACHE II scores, and a longer length of hospital and ICU stay^23,24^. Prolonged hospitalization leads to decreased physical activity, while worsening illness severity (such as systematic inflammation, mechanical ventilation, exposure to glucocorticoids, or neuromuscular blocking agents) increases the risk of ICU-AW. Our study indicated StO_2_ would also be associated with ICU-AW.

In this study, the incidence of ICU-AW was 18%. According to a previous study, the incidence of ICU-AW was 50%^2^, which is more frequent than that in our study population. This may be because we recruited various types of patients, including postsurgical patients. Almost half of our study population had abdominal emergencies, which required a shorter duration of mechanical ventilation after surgery. Regarding ultrasonography measurements, long-term studies have shown that an increased stay in the ICU is associated with a substantial reduction in muscle thickness and CSA of the RF (approximately 30% on day 10 after ICU admission)^29^. In our study, the RF thickness and CSA significantly decreased after seven hospitalization days (a decrease of approximately 20% from admission). This is a very important finding of our study, since it shows that muscle atrophy occurs earlier than what was reported in other studies. This finding highlights the importance of promptly predicting the occurrence of ICU-AW.

However, although StO_2_ is tightly linked with muscle metabolism, low StO_2_ cannot be explained only by an increase in oxygen consumption. As the increase in oxygen consumption leads to an increase in oxygen delivery (supply), even the oxygen consumption increases, it may not be shown as the decrease of StO_2_. On the other hand, a lack of oxygen delivery is the problem as it causes ischemia and likely leads to a muscle atrophy, yet this issue may not be identified as the decrease of StO_2_ if the oxygen consumption is accommodated (decreased). Occlusion Test can provide more detailed information from the downward slope of infrared signals as it artificially stops the oxygen delivery that allows for the assessment of oxygen consumption if it is simultaneously measured by NIRS^30^. However, because the current study did not include the occlusion test, we cannot make a clear statement of which mechanisms attributed to the lowest number of StO_2_ (nadir StO_2_). This will be further investigated in the next stage of our project.

According to the results of the multivariate analysis, StO_2_ was selected as an independent factor associated with MRC-SS, while IL-6 was not. Studies have reported a strong relationship between low StO_2_ and a worse morbidity^7–9^. One study showed that StO_2_ could be a better predictor than other classic parameters^7^. When adjusted for severity and length of hospitalization, StO_2_ was a better predictor for MRC-SS than IL-6. Due to the aforementioned reason, we cannot explain the mechanism of how this occurs. However, the possible explanation is that StO_2_ can measure local muscle function^31–33^ but IL-6 represents the systemic level of inflammatory change and/or metabolism. Further mechanistic studies may warrant to be investigated.

Several studies have investigated indicators that predict the development of ICU-AW. According to Wiske et al.^22^, three easily available parameters (lactate levels, treatment with aminoglycosides, and age) had fair discriminative performance for detecting ICU-AW. Mitobe et al.^34^, reported the relationship between the presence of ICU-AW and the CSA of the skeletal muscle at the level of the third lumbar vertebra using abdominal computed tomography scans on admission In addition, several interventions are considered useful for preventing ICU-AW. According to a previous meta-analysis, early mobilization appears to decrease the incidence of ICU-AW^35^. Intervention with neuromuscular electrical stimulation can also improve muscle strength during hospitalization^36^. However, interventions aimed at attenuating the catabolic state during the acute phase of hospitalization are likely to have the greatest effect by preventing, rather than restoring, the subsequent impact on mass and function. Further investigation is needed to clarify which indicator can predicts future complications the most.

This study has several limitations. First, the sample size was relatively small. Therefore, they could not be grouped according to the disease or severity. In patients with severe morbidity such as sepsis, a decreased StO_2_ may be observed. Changes over time in the variables related to StO_2_ would differ depending on the patient’s outcome (such as discharge to home, transfer to a different hospital, and death). Moreover, we could not analyze the relation between muscle atrophy and other factors such as neuromuscular blocking agents. However, no matter what the diseases or outcomes were, the patients in our study population had very low StO_2_ during the hospitalization as compared to the normal reference value. The normal value of StO_2_ at the vastus lateralis was reported as 74.4% (68.6–80.1) demonstrated by the previous study, in which the values were obtained from healthy individuals^37^. Due to the nature of our study design (observational), it was difficult to measure the variables before patient’s admission. This is one of the limitations of our study that we do not have data from our own healthy individuals or internal control values from our patients. In addition, due to a limited number of patients, we were not able to perform sex-oriented analysis. In our study population, male (59%) was more than female (41%). However, the stepwise-selecting process of the multivariate analysis dropped the gender from independent factors, meaning that sex differences seemed not associated with MRC-SS. A comprehensive exploration of sex-specific mechanisms is beyond the scope of this study, but owing to the importance of sex differences in clinical practice and a need for understanding of their basic mechanisms, future studies may warrant the investigation of sex differences. We used a convenient sample of critically ill patients, which may not be truly representative of the general ICU population. Therefore, further investigations with multi-center and large cohort will be warranted. Secondly, we did not undergo vascular occlusion test, which is the one way to assess O_2_ delivery and oxygen consumption by using StO_2_^30^. Thus, we could not exclude the possibility of low tissue perfusion in the presence of normal tissue oxygen consumption. We selected the method which was more applicable to any clinical settings. The next step of this study is to obtain more mechanistic data by NIRS and so the further investigation will be conducted. Finally, we could not exclude the effect of a tissue edema on the ultrasonography measurements. It has been debated as the main issue of using the ultrasonography to critical illness patients. In order to assess the tissue edema, using bioimpedance assessments (BIA) is preferred. However, according to the previous study, patients who are affected by the muscle edema, tend to increase their muscle mass from days 1 to 7 when measured by the ultrasonography^26^. However, most our patients demonstrated opposite, showing a decreasing trend through day 7, which may suggest that the muscle atrophy was predominant in our patients despite a potential effect from the muscle edema.

In conclusion, StO_2_ of the quadriceps muscle may be useful in predicting muscle atrophy and dysfunction in critically ill patients. StO_2_ is easily and noninvasively measurable at bedside in ICU settings; therefore, it can be a critical parameter for predicting ICU-AW. Early prediction may prevent ICU-AW by providing appropriate intervention before the event.

### Supplementary Information

Supplementary Legends.

Supplementary Figures.

Supplementary Tables.

## Data Availability

The datasets used and/or analyzed during the current study available from the corresponding author on reasonable request.

## Abbreviations

CSA: Cross-sectional area
deoxy-Hb: Deoxyhemoglobin
ICU: Intensive care unit
ICU-AW: Intensive care unit-associated muscle weakness
IL-6: Interleukin-6
MRC: Medical Research Council
MRC-SS: MRC scale scores
NIRS: Near-infrared spectroscopy
oxy-Hb: Oxyhemoglobin
RF: Rectus femoris
StO_2_: Tissue oxygen saturation
