# Supplementary material for: Association of tissue oxygen saturation levels with skeletal muscle injury in the critically ill
Source: Sci Rep. 2024 Feb 27;14:4811. doi: 10.1038/s41598-024-55118-1 (PMC10899231; doi:10.1038/s41598-024-55118-1)
Supplement: Supplementary file 1 — Supplementary Legends. [file 41598_2024_55118_MOESM1_ESM.docx]

**Supplementary figure captions**

**Supplementary Fig.1 Ultrasound scan of the rectus femoris using trapezoid scan.**

(a) The muscle thickness was measured from the lower to the upper part of the rectus femoris muscle above the femur (b) The cross-sectional area (CSA) and the regions of interest (ROI) for echo intensity measurements were drawn within the muscle following the contours of the muscle just below the fascia. We used “trapezoid scan” mode to expand the view of the linear transducer to obtain the entire CSA in the ultrasound window.

**Supplementary Fig.2 Relative change in ultrasound measurements between day 1 and day 7 after admission.**

RF: rectus femoris, CSA: cross-sectional area

Red dots indicate the relative increase, i.e., swelling. Blue dots indicate relative decrease, i.e., atrophy. Red dots line indicates that there was no change between day 1 and day 7.
