# Supplementary figures and images for: Association of tissue oxygen saturation levels with skeletal muscle injury in the critically ill
Source: Sci Rep. 2024 Feb 27;14:4811. doi: 10.1038/s41598-024-55118-1 (PMC10899231; doi:10.1038/s41598-024-55118-1)

## Slide 1
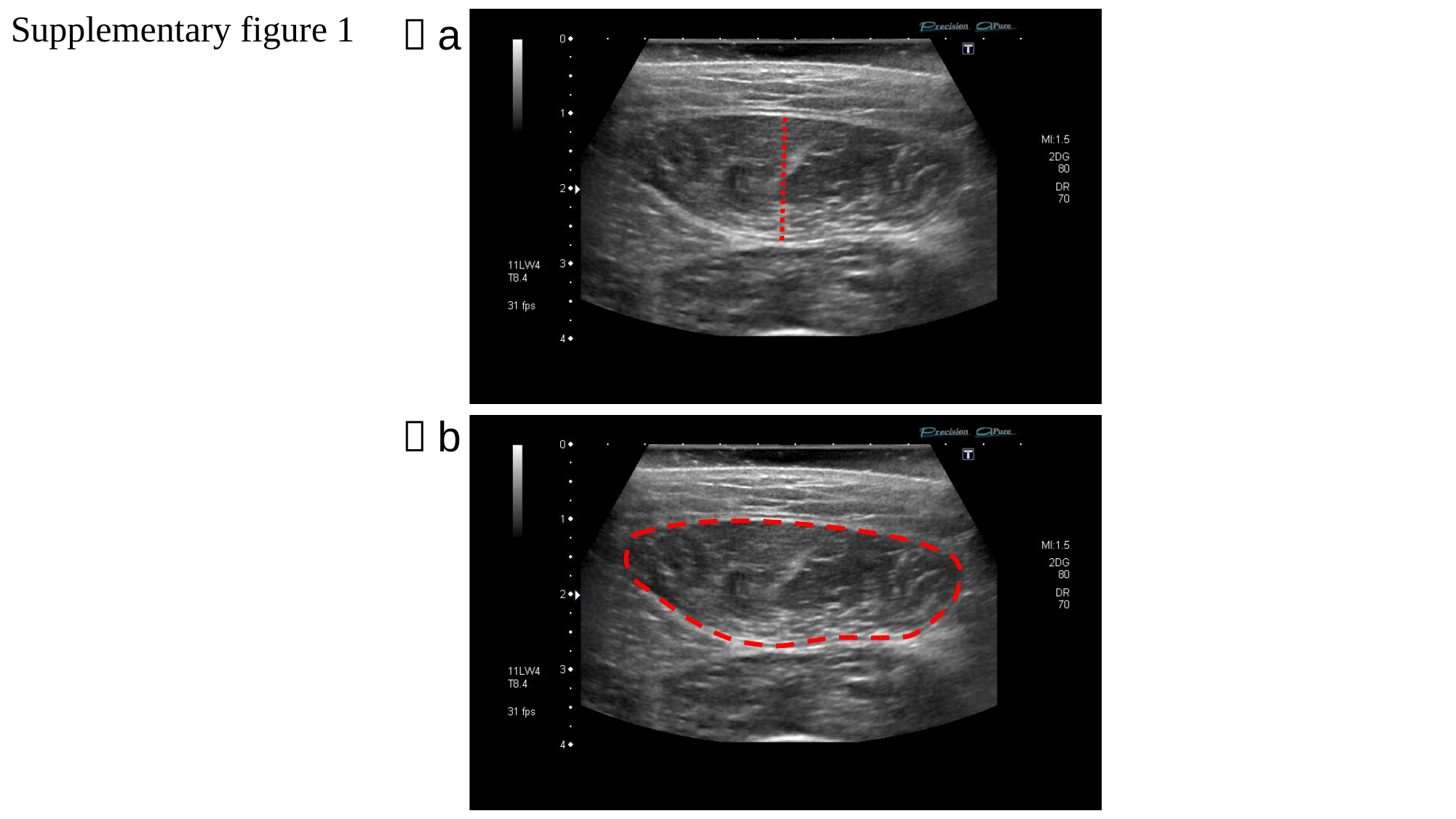

Supplementary figure 1
（a）
（b）

## Slide 2
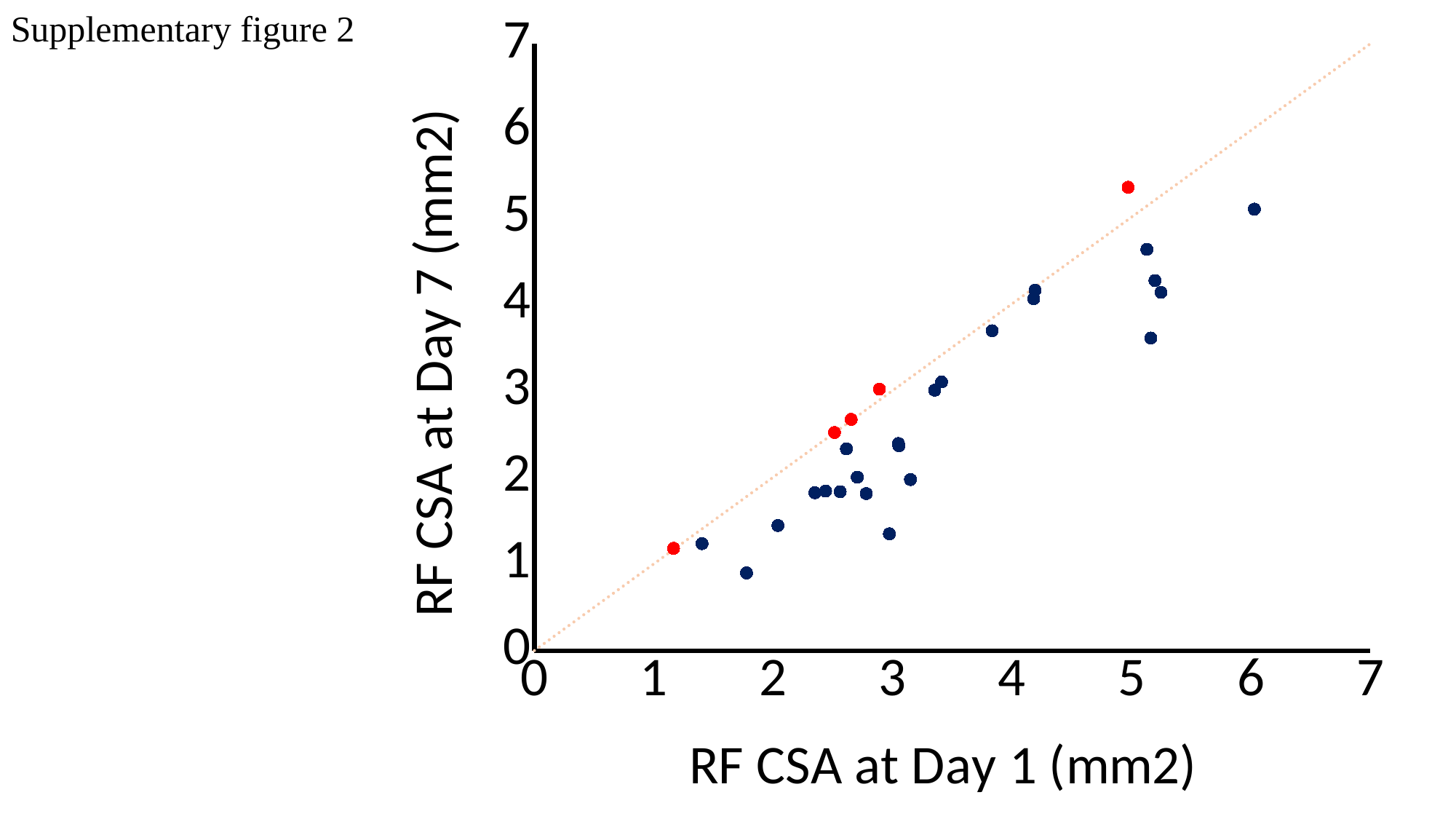

Supplementary figure 2
### Chart
| Category | | | |
|---|---|---|---|

Supplement: Supplementary file 2 — Supplementary Figures. [file 41598_2024_55118_MOESM2_ESM.pptx]
