# Supplementary material for: Association of tissue oxygen saturation levels with skeletal muscle injury in the critically ill
Source: Sci Rep. 2024 Feb 27;14:4811. doi: 10.1038/s41598-024-55118-1 (PMC10899231; doi:10.1038/s41598-024-55118-1)
Supplement: Supplementary file 3 — Supplementary Tables. [file 41598_2024_55118_MOESM3_ESM.docx]

**Supplementary Tables**

**Supplementary Table S1. Correlation between each blood parameter values and other variables**

|  | **Coefficient correlation (r)** | | | | |
| --- | --- | --- | --- | --- | --- |
| **Variables** | MRC-SS | Nadir StO_2_ | Changes in RF thickness | Changes in RF CSA | Changes in RF EI |
|  |  |  | (admission to ICU --> discharge from ICU) | | |
| CRP (mg/L) | -0.20 | -0.33 | -0.09 | 0.16 | -0.22 |
| WBC (×103/mm3) | 0.15 | 0.18 | 0.22 | -0.08 | 0.15 |
| Albumin (g/dL) | -0.35 | -0.19 | -0.21 | -0.01 | 0.14 |
| LDH (U/L) | 0.13 | 0.10 | -0.12 | -0.03 | 0.24 |
| Lactate (mg/dL) | 0.01 | -0.11 | -0.24 | -0.05 | 0.07 |

*: p<0.05, **: p<0.01

CRP: C-reactive protein, WBC: white blood cell, Alb: albumin, LDH lactate dehydrogenase, MRC-SS: Medical Research Council sum score, StO_2_: tissue oxygen saturation, RF: rectus femoris, CSA: cross-sectional area, EI: echo intensity

**Supplementary Table S2. Correlation between MRC-SS and other variables**

|  | **Coefficient correlation (r)** |  |
| --- | --- | --- |
| **Variables** | **MRC-SS** | ***p*-value** |
| Age (years) | 0.19 | 0.35 |
| Length of hospital stay (days) | -0.59 | <0.01 |
| APACHE Ⅱ score (points) | -0.54 | <0.01 |
| log IL-6 (pg/mL) | -0.53 | <0.01 |
| Nadir StO_2_ (%) | 0.41 | 0.03 |
| RF thickness at admission (cm) | 0.24 | 0.23 |
| RF CSA at admission (cm^2^) | 0.06 | 0.78 |

MRC-SS: Medical Research Council sum score, APACHE: Acute Physiology and Chronic Health Evaluation, IL-6: Interleukin-6, StO_2_ tissue oxygen saturation

**Supplementary Table S3. Multiple regression models for prediction of MRS-SS**

|  | **Model 1** | | **Model 2** | | **Model 3** | | **Model 4** | |
| --- | --- | --- | --- | --- | --- | --- | --- | --- |
| **Variables** | **β** | ***p*-value** | **β** | ***p*-value** | **β** | ***p*-value** | **β** | ***p*-value** |
| Length of hospital stay (days) | -0.33 | 0.06 | -0.39 | <0.01 | -0.34 | 0.08 | † |  |
| APACHE Ⅱ score (points) | -0.43 | <0.01 | -0.45 | <0.01 | -0.34 | 0.05 | † |  |
| Nadir StO_2_ (%) | 0.38 | 0.02 | 0.38 | <0.01 | † |  | -0.46 | 0.02 |
| log IL-6 (pg/mL) | -0.15 | 0.41 | † |  | -0.27 | 0.17 | 0.26 | 0.16 |
| Adjusted R^2^ of the model | 0.54 |  | 0.58 |  | 0.42 |  | 0.28 |  |

MRC-SS: Medical Research Council Sum Score, APACHE: Acute Physiology and Chronic Health Evaluation, StO_2_ tissue oxygen saturation

^†^Variables not included in the model

**Supplementary Table S4. Multiple regression models for independent prediction of RF thickness**

| **Variables** | **β** | ***p*-value** | **Adjusted R^2^** |
| --- | --- | --- | --- |
| log IL-6 (pg/mL) | -0.57 | <0.01 | 0.31 |
| Age (years) | † |  |  |
| Gender | † |  |  |
| Length of hospital stay (days) | † |  |  |
| APACHE Ⅱ score (points) | † |  |  |
| Nadir StO_2_ (%) | † |  |  |

RF: rectus femoris, IL-6: Interleukin-6, APACHE: Acute Physiology and Chronic Health Evaluation, StO_2_ tissue oxygen saturation

^†^Variables not included in the model

**Supplementary Table S5. Multiple regression models for prediction of RF CSA**

| **Variables** | **β** | ***p*-value** | **Adjusted R^2^** |
| --- | --- | --- | --- |
| Length of hospital stay (days) | -0.49 | <0.01 | 0.22 |
| Age (years) | † |  |  |
| Gender | † |  |  |
| APACHE Ⅱ score (points) | † |  |  |
| log IL-6 (pg/mL) | † |  |  |
| Nadir StO_2_ (%) | † |  |  |

RF: rectus femoris, CSA: cross-sectional area, APACHE: Acute Physiology and Chronic Health Evaluation, StO_2_ tissue oxygen saturation

^†^Variables not included in the model
